# Dr. T. B. Welch

**Published:** 1904-02

**Authors:** 


### Obituary.

### DR. T. B. WELCH.

Died, at Overbrook, near Philadelphia, Pa., December 29, 1903, Thomas Bromwell Welch, M.D., aged seventy-eight years.

Dr. Welch was born in Glastonbury, Somersetshire, England, December 31, 1825, and came to this country when quite young. His education was obtained at the public schools of Watertown, N. Y. After a course of study at the Gouverneur Wesleyan Seminary, he entered the Wesleyan Methodist ministry, and served several charges in New York State. Later he entered a medical college and graduated with the medical degree when in his twenty-sixth year. After a few years' medical practice he decided to practise dentistry, settling in Winona, Minn., about 1856. In 1865 he removed to Vineland, N. J., where he continued dental practice. About 1869 he originated a method of preserving grape-juice absolutely free from alcohol, especially designed for church communion and medical uses. This grape-juice became more and more popular as the years went by, and is now well known all over the world.

Dr. Welch was first brought prominently to the notice of the dental profession as the manufacturer of an improved alloy for amalgam. Shortly after the late Dr. Flagg and his colaborers had announced their "New Departure" theories, there was a demand in the profession for something better than the alloys then on sale at the dental depots. Dr. Welch had prepared an alloy for his own use, samples of which he distributed to some of his professional friends, who were much pleased with it. This was brought to the notice of the New Jersey State Dental Society, of which Dr. Welch was then a member, at the meeting held July, 1878, and resulted in a resolution requesting Dr. Welch, or any other of its members, to place on the market an alloy, reliable, and yet at a reasonable price. In response to this, Dr. Welch began the manufacture of his Gold-and-platina alloy; and that it was "reliable" is attested by its quickly becoming, and still remaining, notwithstanding its many competitors, the stand-by of many excellent operators.

At the same time he began the publication of a bi-monthly dental journal, mainly as an advertising medium, entitled *Items of Interest.* This first appeared as a four-page folio, June, 1879. As its name indicated, it was made up of "items of interest" to dentists, and as Dr. Welch possessed the rare, yet happy faculty of quickly discerning, condensing, and properly presenting items of interest appearing in dental and other journals, his unpretentious little journal soon made for itself a place in dental literature. The second volume terminated with the December number (No. 4), 1880, in order to begin Vol. III. with January, 1881; and with the third number, May, 1881, it became, and has since continued, a monthly. Beginning with Vol. IV. the number of pages was increased to sixteen, and their size reduced from folio to quarto. The first number of Vol. V. was issued in quarto form, but with the

February number it was changed to an octavo of forty-eight pages, and later the January number was reissued to correspond. The yearly subscription of Vols. I. and II. was fifteen cents, Vols. III. and IV., fifty cents, and with Vol. V. it was raised to one dollar. While still at Vineland, Dr. Welch enlarged his business to include dental supplies, and early in 1881 removed to 1405 Filbert Street, Philadelphia, where, under the firm name of Drs. T. B. Welch & Son, he opened a dental depot. In a few months he moved to 1413 Filbert Street; in June, 1885, the firm-name became "Welch Dental Company," and this, in March, 1889, gave way to the "Wilmington Dental Manufacturing Company." In July, 1896, this corporation became financially embarrassed and terminated, and the journal Dr. Welch had so long edited passed into other hands. Dr. Welch then began the publication of a dental journal in much the same style entitled *Welch's Dental Journal,* which later, in other hands, became the *Dental Brief.* Dr. Welch was a ready writer. He earnestly urged the importance of terseness and clearness upon contributors to his journal, and set a good example in these respects by his own contributions.

Dr. Welch was a strong advocate of total abstinence, and took an active part in the work of seeking out and prosecuting illegal liquor selling. He was also interested in the so-called spelling reform, substituting phonetic, or sound spelling, for the arbitrary method in general use.

Dr. Welch possessed a kindly disposition, and delighted in doing good. He was a devoted and prominent member of the Methodist church in Vineland, where he so long resided, and where he was buried.

After the death of his wife he married, in 1895, Miss Sherburne, of Vineland, who survives him. His surviving children are Drs. George and Charles E. Welch; Mrs. Dr. Emma Slade, Mrs. Dr. Thomas, Mrs. Professor Murray, and Mrs. Milo Gould.

